# Supplementary material for: The phospho-ferrozine assay: a tool to study bacterial redox-active metabolites produced at the plant root
Source: Appl Environ Microbiol. 2024 Dec 17;91(1):e02194-24. doi: 10.1128/aem.02194-24 (PMC11784245; doi:10.1128/aem.02194-24)
Supplement: Supplemental figures — Figures S1 to S8. [file aem.02194-24-s0001.pdf]

Supplemental Figures

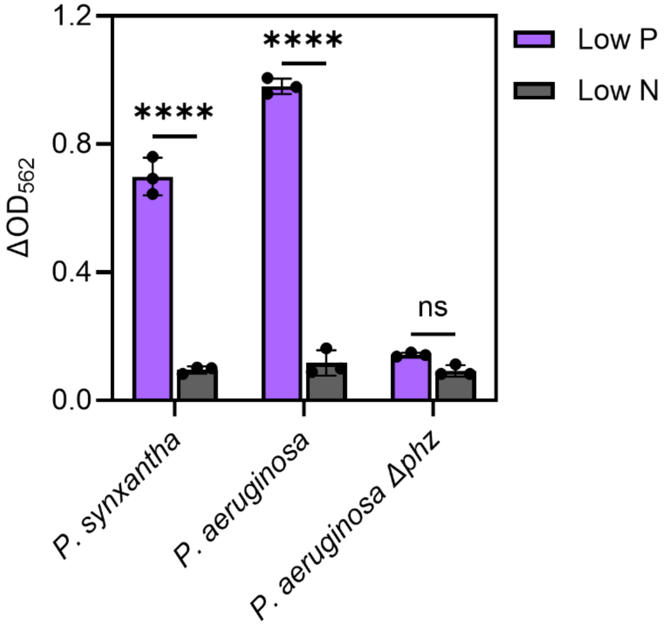

Figure S1. The ferrozine assay detects production of redox-active metabolites in response to P-limitation by *P. synxantha* and *P. aeruginosa*. Low P = P-limitation (0.1 mM). Low N = N-limitation (2 mM). Data shown are means ± SD from three biological replicates. Statistical significance was determined by a Two-Way ANOVA test comparing the ferrozine signal of the Low P to the Low N condition within each strain. ns = not significant; **** = $p < 0.0001$.

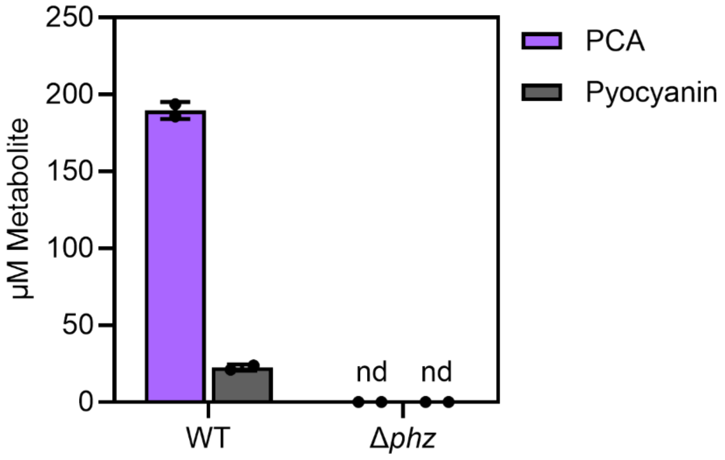

Figure S2. Liquid chromatography-mass spectrometry data of P-limited *P. aeruginosa* filter-sterilized supernatants showing that the phenazine PCA is more highly produced than pyocyanin under our media conditions (mixture of glucose, pyruvate, and succinate as carbon sources). Data shown are the means ± SD of two biological replicates. nd = not detected.

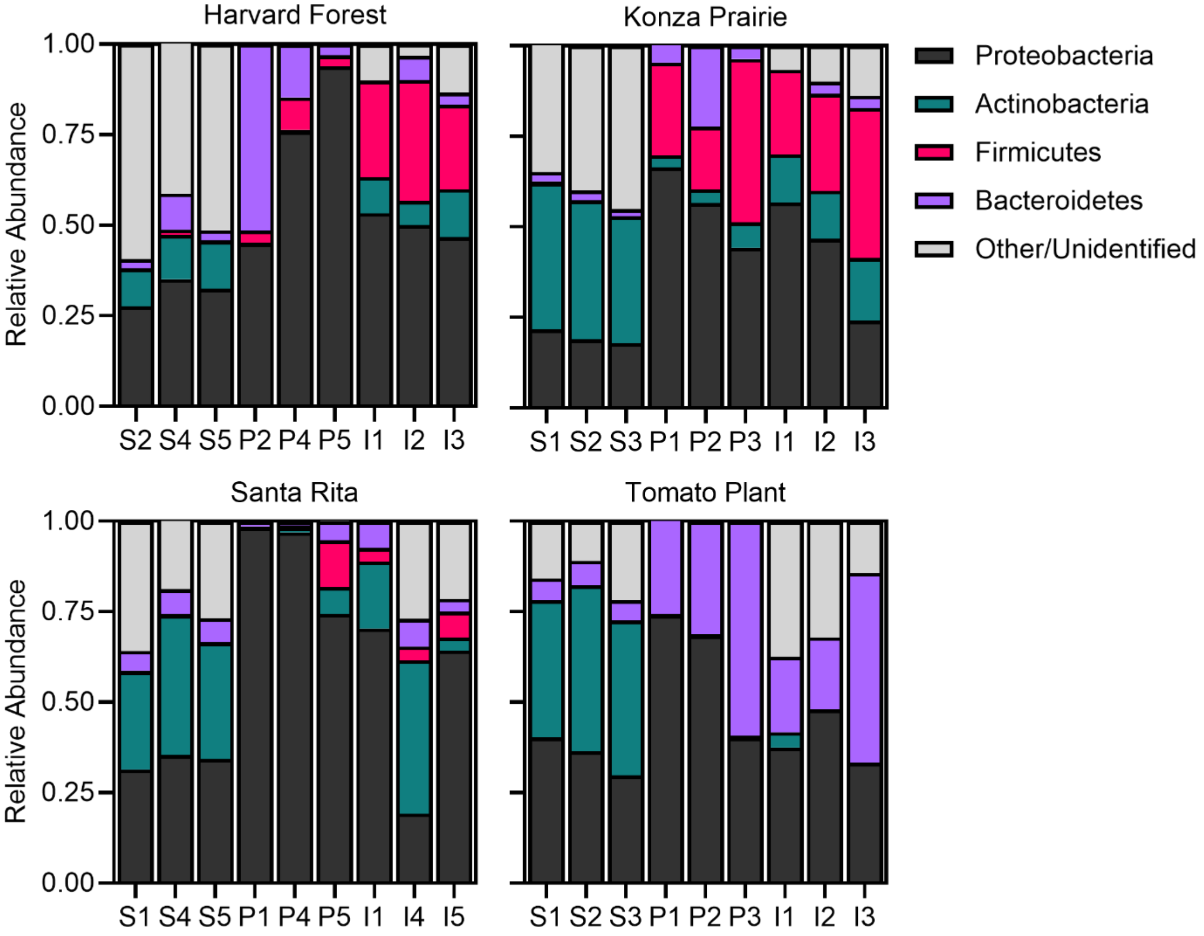

Figure S3. Taxonomic distribution of bacteria from root-associated soil, yeast extract agar plates, and purified isolates. Each plot depicts the taxonomic diversity of three plants from each site. Numbers correspond to a different plant analyzed and 16S amplicon sequencing was performed to determine the taxonomic diversity from soil and yeast extract plates. The choice of plant for analysis was arbitrary. The diversity of purified isolates was determined by colony PCR. S = soil; P = yeast extract plates; I = Purified isolates.

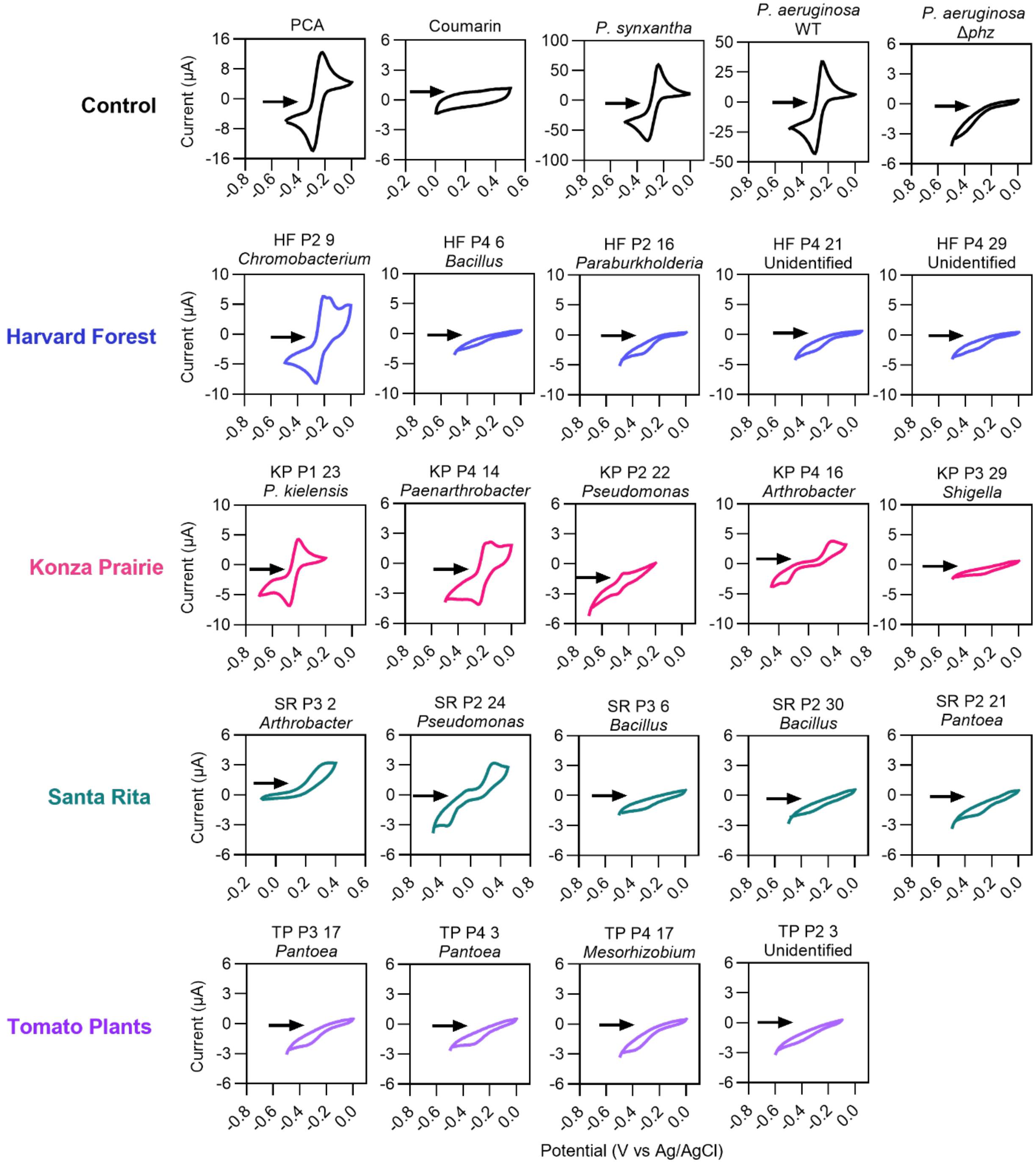

Figure S4. Voltammograms of filter-sterilized supernatants from P-limited soil isolates that showed an increased ferrozine signal relative to *P. aeruginosa* Δ*phz* (Fig. 4C). Scans shown are the second of three scans using a gold working electrode. Colors correspond to sites where isolates were obtained.

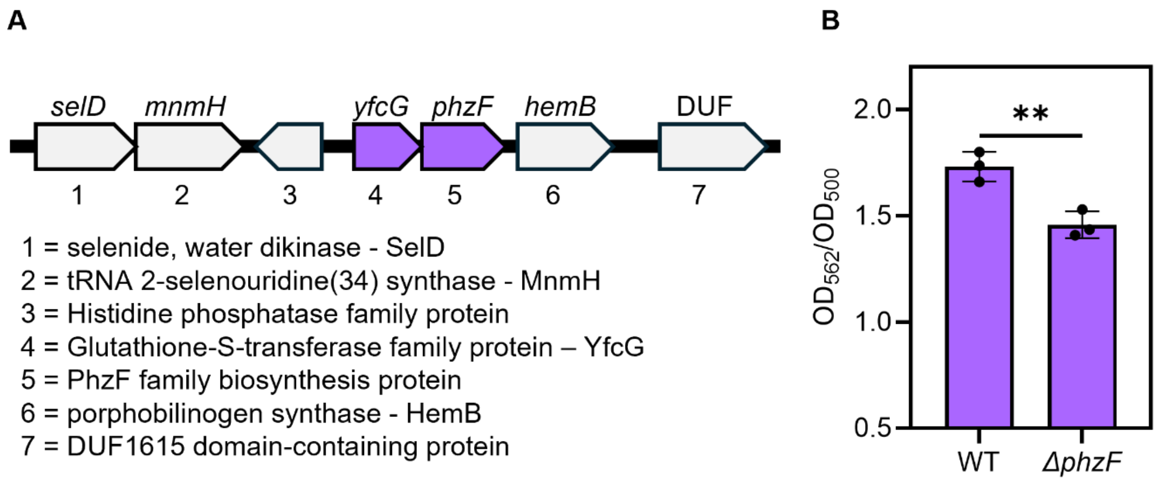

Figure S5. PhzF is not a major contributor to RAM production. (A) Genetic cluster of *phzF* and its neighboring genes. *phzF* is predicted to be in a two-gene operon with *yfcG* (purple). **(B)** Deletion of *phzF* slightly decreases phospho-ferrozine signal of P-limited *P. kielensis* supernatants. Phospho-ferrozine signal (OD_{562}) of *P. kielensis* is normalized to bacterial growth (OD_{500}). Data shown are the means ± SD from three biological replicates. Statistical significance was determined with an unpaired t-test. ** = $p < 0.01$.

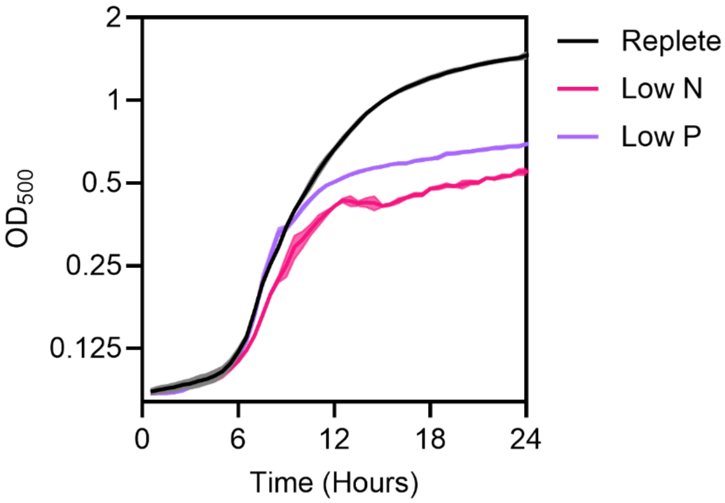

Figure S6. *P. kielensis* growth is limited in Low N (2 mM nitrogen) and Low P (0.1 mM phosphorus) media compared to the replete media (16 mM nitrogen/7 mM phosphorus). Data shown are means ± SD of three biological replicates.

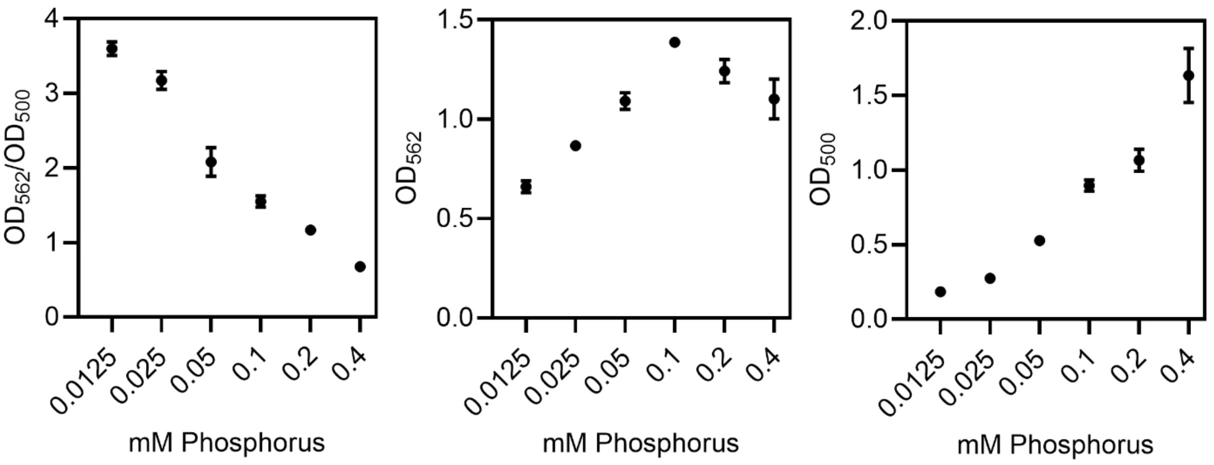

Figure S7. Redox-active metabolite biosynthesis by *P. kielensis* is dependent on the amount of phosphorus available. *P. kielensis* was grown across a range of P concentrations (0.0125 to 0.4 mM) for 24 hours, and filter-sterilized supernatants were used for the phospho-ferrozine assay. Data show that increasing P lowers growth normalized redox-active metabolite biosynthesis (as inferred from the ferrozine assay) by *P. kielensis* (left panel). Right panel shows that increased phosphorus increased the final cell density after 24 hours (OD_{500}). Middle panel shows that increasing phosphorus also increases the ferrozine signal (OD_{562}, measured after the addition of Fe and ferrozine at the end of growth) but that the signal does not keep pace with growth and instead decreases above 0.1 mM P. Left most panels shows growth normalized ferrozine signal (middle panel normalized to right panel). Data shown are means ± SD of three biological replicates.

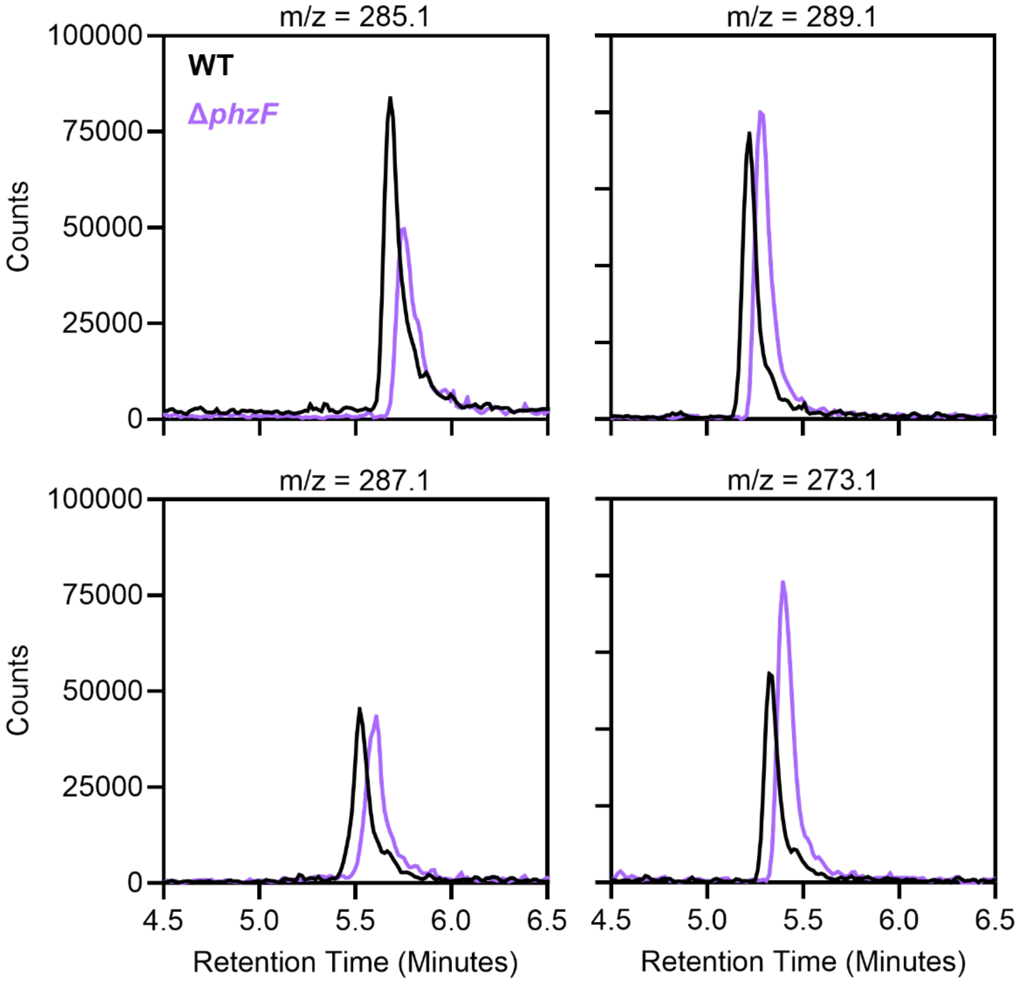

Figure S8. Deletion of *phzF* does not abolish metabolite production. Data show that Extracted Ion Chromatograms (EIC) from P-limited supernatants of WT and Δ*phzF* mutants are similar. EICs are for four masses shown to be present in P- not but N-limited *P. kielensis* supernatants (Fig. 5).
